# Spatial and seasonal distribution of human schistosomiasis intermediate host snails and their interactions with other freshwater snails in 7 districts of KwaZulu-Natal province, South Africa

**DOI:** 10.1038/s41598-023-34122-x

**Published:** 2023-05-15

**Authors:** Onyekachi Esther Nwoko, Tawanda Manyangadze, Moses John Chimbari

**Affiliations:** 1 Discipline of Public Health Medicine, College of Health Sciences, University of KwaZulu-Natal, Durban, 4000 South Africa; 2 Geosciences Department, School of Geosciences, Disaster and Development, Faculty of Science and Engineering, Bindura University of Science Education, Bag 1020, Bindura, Zimbabwe; 3 Department of Behavioural Science, Medical and Health Sciences, Great Zimbabwe University, P.O Box 1235, Masvingo, Zimbabwe

**Keywords:** Freshwater ecology, Diseases

## Abstract

The spatial and seasonal distribution, abundance, and infection rates of human schistosomiasis intermediate host snails and interactions with other freshwater snails, water physicochemical parameters, and climatic factors was determined in this study. A longitudinal malacology survey was conducted at seventy-nine sites in seven districts in KwaZulu-Natal province between September 2020 and August 2021. Snail sampling was done simultaneously by two trained personnel for fifteen minutes, once in three months. A total of 15,756 snails were collected during the study period. Eight freshwater snails were found: *Bulinus globosus* (n = 1396), *Biomphalaria pfeifferi* (n = 1130), *Lymnaea natalensis* (n = 1195), *Bulinus tropicus* (n = 1722), *Bulinus forskalii* (n = 195), *Tarebia granifera* (n = 8078), *Physa acuta* (n = 1579), and Bivalves (n = 461). The infection rates of *B. globosus* and *B. pfeifferi* are 3.5% and 0.9%, respectively. In our study, rainfall, pH, type of habitats, other freshwater snails and seasons influenced the distribution, abundance, and infection rates of human schistosomiasis intermediate host snails (*p-*value < 0.05). Our findings provide useful information which can be adopted in designing and implementing snail control strategies as part of schistosomiasis control in the study area.

## Introduction

Neglected tropical diseases (NTDs) including schistosomiasis are a group of preventable and treatable communicable diseases that affect over 1.7 billion people around the world^1^. These diseases are commonly found in tropical and sub-tropical countries or regions with inadequate health care, scarce clean water, and sanitation worsened by climate change. Schistosomiasis, which presents the second highest burden among NTDs, affects about 30 million people worldwide, with about 93% of the infected people living in African regions^2^. In South Africa, it is estimated that about 5.2 million people need treatment for schistosomiasis. KwaZulu-Natal (KZN) province is one of the worst affected areas, with a prevalence between 22 and 55% among children^3^. Freshwater snails are essential for the transmission of schistosomiasis as they serve as intermediate hosts^4^.

Schistosomiasis is caused by trematode worms of the genus *Schistosoma* being transmitted through intermediate host snails. Schistosomiasis has two major forms; intestinal schistosomiasis and urogenital schistosomiasis, caused by 5 main species of schistosomes; *Schistosoma mansoni*, *S. haematobium*, *S. japonicum*, *S. intercalatum,* and *S. mekongi*. Urogenital schistosomiasis is caused by *S. haematobium,* while intestinal schistosomiasis is caused by other species^5^. In South Africa, *S. haematobium* and *S. mansoni* are the common species of human schistosomiasis^6^. *S. haematobium* and *S. mansoni* are mainly transmitted by the *Bulinus* and *Biomphalaria* snail genus, respectively. When human faeces or urine with parasitic eggs are released into freshwater, the eggs hatch into miracidia that search for and penetrate appropriate intermediate host snails. Asexual reproduction takes place in the snails resulting in the evolution of miracidia into sporocyst. Sporocysts grow into cercariae that get released into water bodies and penetrate human skin to complete the cycle and cause the disease^5^.

Other freshwater snails such as *Bulinus tropicus*, *Bulinus forskalii*, *Physa acuta*, *Tarebia granifera,* and Bivalves, which are not intermediate host for human schistosomiasis have to a lesser extent been studied in KwaZulu-Natal compared to human schistosomiasis intermediate host snails: *Bulinus globosus* and *Biomphalaria pfeifferi*^7^. Previous studies have shown the effects of water physicochemical parameters such as water temperature, pH, dissolved oxygen, total dissolved solids, electrical conductivity, and climatic factors such as land surface temperature and rainfall on the distribution and abundance of freshwater snails^6,7^.

A cross-sectional study on the diversity, distribution and abundance of freshwater snails in all the districts in KZN province has also been carried out^7^. However, the study lacked information on temporal and seasonal patterns of freshwater snails in KZN as the study was cross-sectional based and accounted for the rainy season only. Thus, our study aimed to (a) identify the spatial and seasonal distribution of intermediate host snails for schistosomiasis in 7 districts in KZN in different seasons, (b) assess the effects of water physicochemical parameters, climatic factors, and other snail species on abundance of intermediate host snails for and (c) determine the infection status of intermediate host snails for schistosomiasis across districts and seasons. World Health Organization (WHO) targets to eliminate schistosomiasis as a public health problem in all endemic countries and interrupt schistosomiasis transmission in humans in selected countries by 2030. As a contribution to this effort, our study identified seasons in which snail distribution, abundance, and infection rates are highest thus providing critical information for designing and implementing of snail control in KZN, South Africa.

## Materials and methods

### Study area

**Figure 1:**
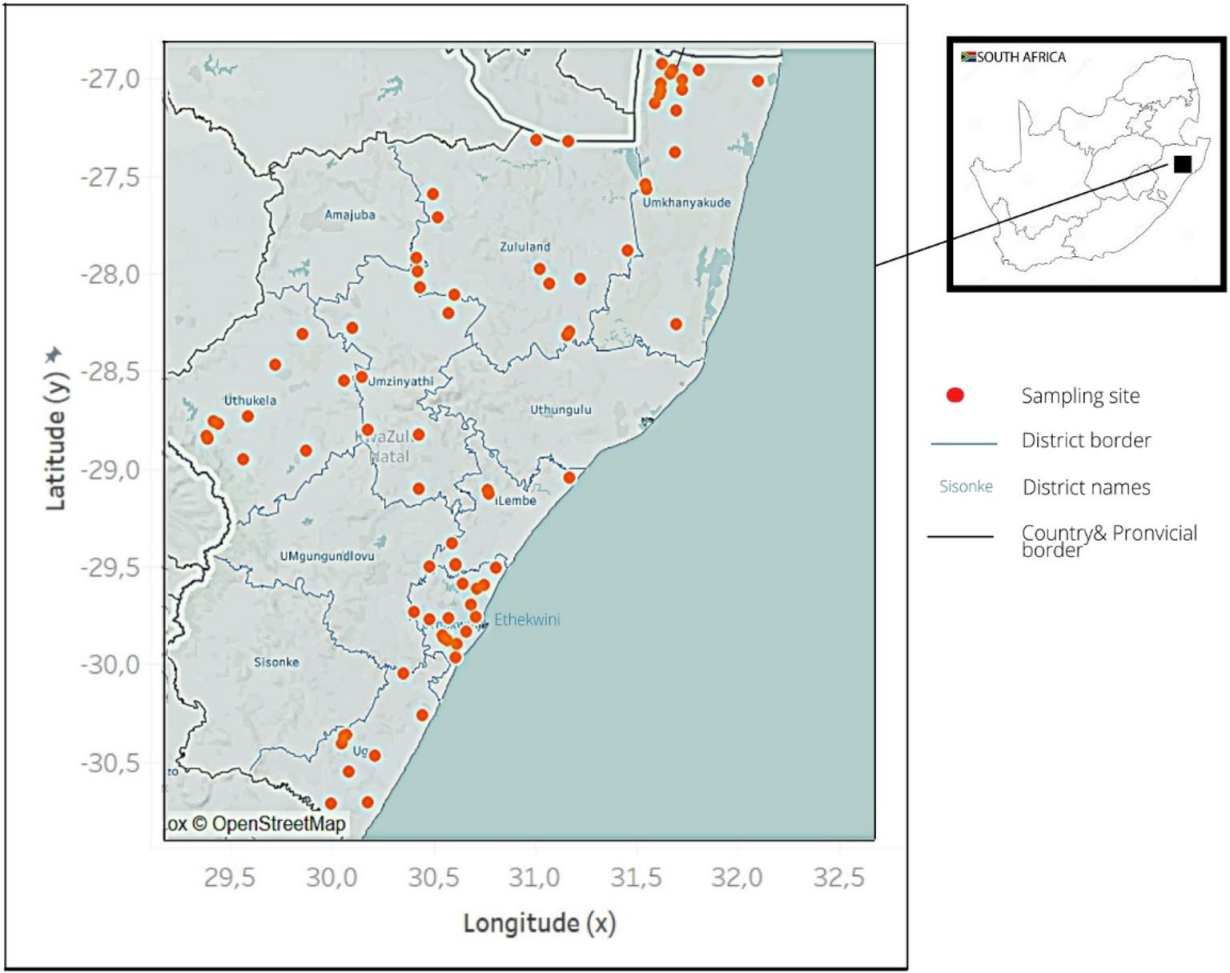
Study area and sampling sites (indicated by red points) in KwaZulu-Natal, South Africa (September 2020 to August 2021).

KZN province experiences four seasons; the hot/dry (September–November), rainy (December–February), post-rainy (March–May), and cold/dry (June–August). We carried out our study in randomly selected districts of KwaZulu-Natal; Zululand, uMzinyathi, uMkhanyakude, uThukela, Ugu, iLembe and eThekwini districts (Fig. 1). eThekwini, a metropolitan municipality has a temperate climate with hot and wet summers while the winters are dry. Temperature varies from 14 to 28 °C and is rarely below 11 °C or above 30 °C. The average annual rainfall is 801.1 mm. iLembe district is in the eastern part of KwaZulu Natal province with warm summers and dry winters dry. Temperature varies from 11 to 28 °C and is rarely below 8 °C or above 33 °C. The average annual rainfall is 512.5 mm. uThukela district is in the Western part of KwaZulu Natal province has dry winters and hot summers. Temperature varies from 4 to 29 °C and is rarely below 1 °C or above 34 °C. The average annual rainfall is 688.7 mm. Ugu district is in the Southern part of KwaZulu Natal province characterized by temperate climate without dry season and warm summers. Temperature varies from 15 to 27 °C and is rarely below 13 °C or above 29 °C. The average annual rainfall is 1207.4 mm. Zululand district is in the northern part of KwaZulu Natal province and has a temperate climate with hot summers and dry winters. Temperature varies from 10 to 31 °C and is rarely below 7 °C or above 35 °C. The average annual rainfall is 732.8 mm. uMzinyathi district is in the northern part of KwaZulu Natal province is characterized by dry winters and warm summers. Temperature varies between 3 and 28 °C and is rarely below − 0 °C or above 32 °C. The average annual rainfall is 670.8 mm. uMkhanyakude district is in the northern part of KwaZulu Natal province. It is an arid area and characterized by a hot and humid summer and a cooler and drier winter. Temperature varies from 12 to 31 °C and is rarely below 9 °C or above 35 °C. The average annual rainfall is 676 mm^8,9^.

### Study design

**Figure 2:**
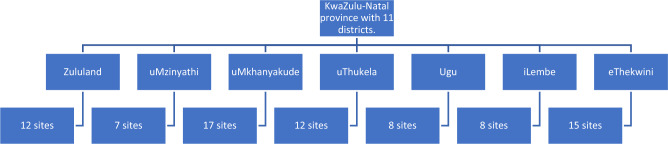
Distribution of sampling sites across districts in KwaZulu-Natal Province.

**Figure 3:**
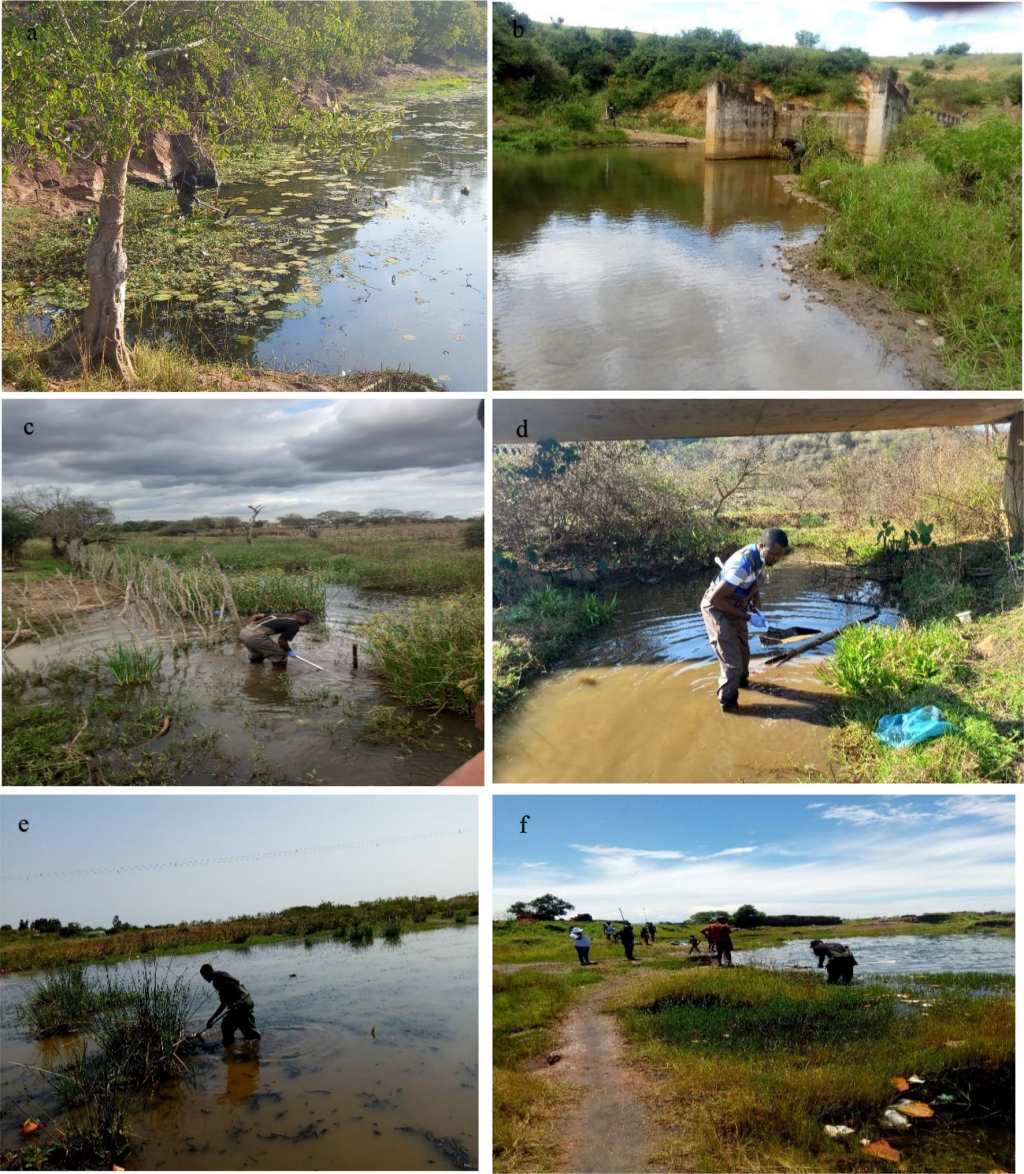
Photographs of some sites (**a**) Mkhayeni stream, (**b**) Ntcoshane stream,** (c**) Mphokathin dam, (**d**) Zamukuzakha stream, (**e**) Embuyeni stream, and (**f**) Shalom dam where snail sampling was carried out in KwaZulu-Natal (KZN) province.

The study was longitudinal and was implemented over 12 months (September 2020–August 2021). Data was collected once during each of the four (4) seasons experienced in KZN province. Seven districts were purposively selected from the 10 districts municipality and 1 metropolitan municipality in KZN province (Fig. 2). The districts were selected based on their location to represent the 4 cardinal points (North, South, East and West). Sampling sites close to schools where parasitological surveys were conducted were selected for the study. Photographs of some sites where snail sampling was carried out in KZN province is presented in Fig. 3.

### Water physicochemical properties and climatic factors

Climatic data was obtained through remote sensing. The Hanna multiparameter meter (HI9829) was used for measuring dissolved oxygen (DO) and pH. Rainfall data was downloaded through the International Research Institute for Climate and Society (IRI) data library (http://iridl.ldeo.columbia.edu/SOURCES/). Minimum and maximum land surface temperature (LST) data were also downloaded (https://app.climateengine.com/climateEngine). The data on water physicochemical parameters were gathered simultaneously with snail surveys. These data were used to determine the effect of climatic factors, water physicochemical parameters, seasons, and habitats on snail distribution, abundance, and infection rates.

### Snail collection and examination of cercarial infection

In each district, survey sites were selected based on proximity to schools where parasitological surveys for another study were conducted and observed human contact activities such as recreation, domestic use, animal watering points, irrigation, and fishing. Freshwater snail sampling was carried out by two experienced and well-trained field collectors using long-handheld scoops, forceps, and handpicking for 15 min. As the scoop was pushed through vegetation; snails were picked out of the scoops by hand using gloves and placed in plastic containers with water and vegetation from the same habitat and then transported to the place for processing. The snails collected were identified morphologically to species level using the standard identification keys developed by Brown and Kristensen^10^. Non-intermediate host snails for schistosomiasis were counted, recorded, and returned to their respective sites. Intermediate host snails for human schistosomiasis were screened for cercarial infections using the cercarial shedding method. Individual snails were placed in vials containing water and exposed to sunlight or artificial light for 1–4 h to induce shedding. The water from the vials were transferred to a petri-dish were stained with iodine solution and placed under a stereomicroscope for identification of cercariae^11^. Snails that did not shed on the first exposure were kept and re-exposed to sunlight after 48 h to induce cercariae shedding. If they still did not shed cercariae, they were crushed to check for developing cercariae or sporocysts^12^. Cercariae was identified morphologically using the key described by Frandsen and Christensen^13^. The Kobo Collect Application (Cambridge, MA, USA) was used to electronically record the data in the field. The data collected includes site name, GPS co-ordinates, habitat type, season, snail species, snail abundance, number of snails shedding mammalian cercariae. Informed consent was obtained to publish the images in Fig. 3 in an online open access publication.

### Statistical analysis

Tableau Public version 2022.2 software^14^, a software used for data visualization and mapping was used to create the map showing the study area and sampling sites. The data included the geographical coordinates, counts of human schistosomiasis intermediate host snails, and names of sites were prepared in Excel spreadsheet and imported to Tableau Public. The software identified the geographic data as well as the descriptive data. Geographic data presented as latitude and longitude were processed by the software and projected on the map of South Africa and we zoomed the map to show the KwaZulu-Natal province only. The descriptive data showing the counts of human schistosomiasis intermediate host snails was also projected on the map in the form circles. The image of the map with the projections of the sites was downloaded and edited using Canva software^15^ to polish the resolution of the map and make the legend which shows the colour representation of the sites, districts and borders.

The data was downloaded from Kobo Collect application into Excel spreadsheet format and analysed in R version 4.1.2^16^. Summary statistics including tables and graphs were used to describe freshwater snails’ abundance, distribution, and infection rates. Kruskal Wallis test is a non-parametric test that was used to statistically compare the difference in the snail abundance and infection rates of freshwater snails among districts^17,18^. The negative binomial generalized linear mixed effect model in the ‘glmmTMB’ package^19^ was used to model the abundance and shedding of *B. globosus* and *B. pfeifferi* snails in relation to climatic factors, water physicochemical properties, habitat types, seasons and other freshwater snails. In the model, site was specified as a random effect to account for variability. Variables with VIF > 5 indicates multicollinearity and were excluded from the current analysis^6^.

### Ethical considerations

This study was approved by the University of KwaZulu-Natal Biomedical Research Ethics Committee (Ref No: BREC/00001305/2020).

## Results

**Figure 4:**
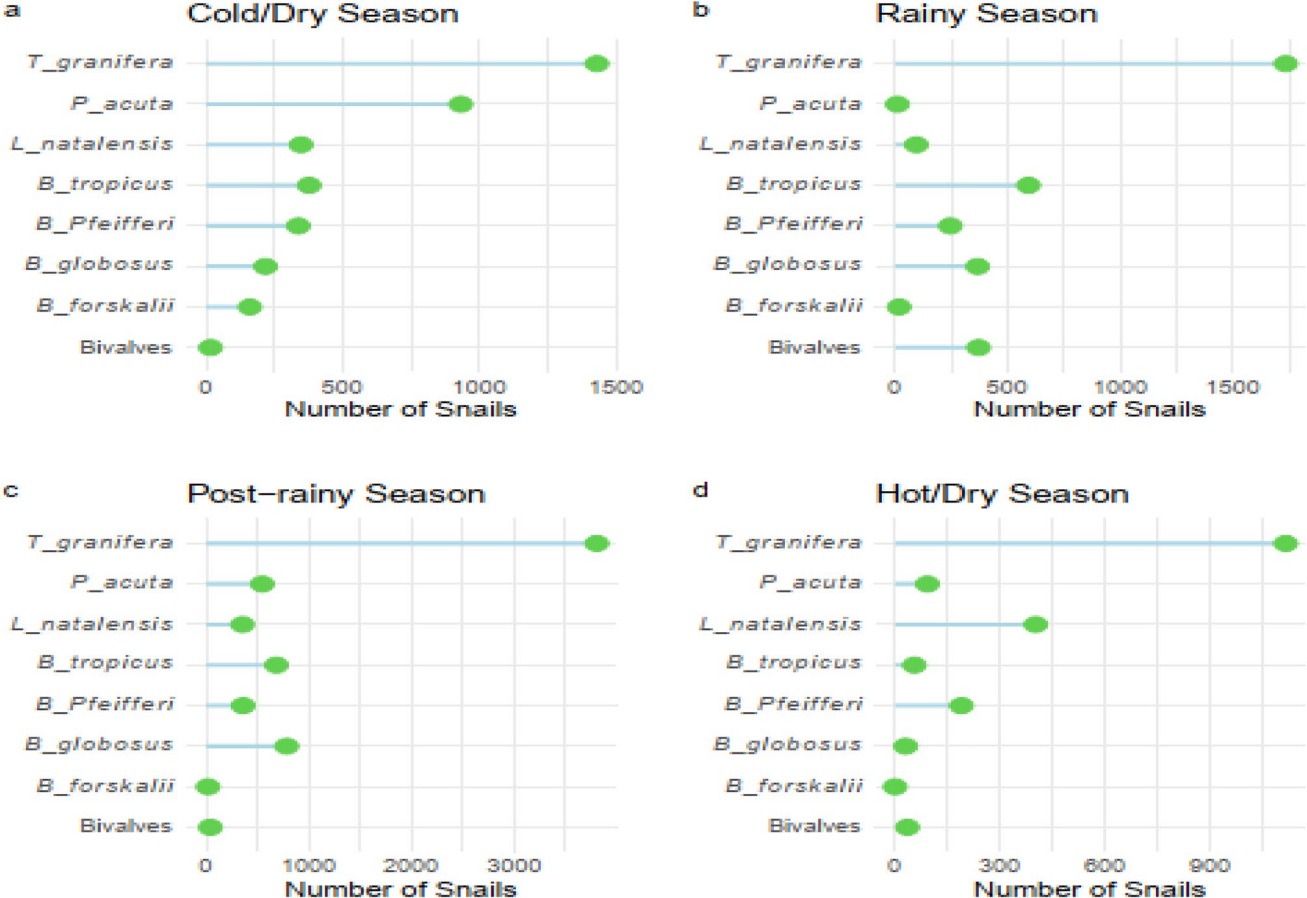
Seasonal distribution and abundance of intermediate host and non-intermediate host snails for human schistosomiasis at sampling sites during 4 seasons.

A total of 15,759 freshwater snails comprising of 8 species were collected in 79 sampling sites in 7 districts in KwaZulu-Natal from September 2020 to August 2021. These snails were identified morphologically. *T. granifera* was the most abundant (n = 8078), followed by *B. tropicus* (n = 1722), *P. acuta* (n = 1579), *B. globosus* (n = 1396), *L. natalensis* (n = 1195), *B. pfeifferi* (n = 1130), Bivalves (n = 461) and *B. forskalii* (n = 195) (Table S1). *B. globosus* was found in 33 sites, *L. natalensis* in 31 sites, *B. tropicus* in 22 sites, *P. acuta* in 21 sites, *T. granifera* in 20 sites, *B. pfeifferi* in 16 sites, Bivalves in 9 sites and *B. forskalii* in 6 sites (Table S1). Most of the freshwater snails were found in streams (n = 64, 81%) while fewer (n = 15, 19%) were found in dams. Overall, snail abundance was highest in the post-rainy season, followed by cold/dry season, rainy season, and hot/dry season (Table S1, Fig. 4). *B. globosus* was found cohabiting with *B. pfeifferi* at 6 sites.

### Effect of districts on snail abundance

The highest and least abundance of *B. globosus* were observed in uThukela (n = 585) and uMzinyathi (n = 46) districts, respectively. Ugu district had the highest abundance (n = 482) of *B. pfeifferi* while iLembe district had the least abundance (n = 1). All the 8 species of snails were found in Ugu and eThekwini districts while only 3 snail species were found in uMzinyathi district. *L. natalensis* and *B. globosus* were found in the 7 districts while Bivalves were only found in 3 districts (Table 1). There was no statistically significant difference in the abundance of *B. globosus* (Kruskal–Wallis ${\chi }_{2.954, df=6, p=0.815}^{2}$), *B. pfeifferi* (Kruskal–Wallis ${\chi }_{8.203, df=6, p=0.224}^{2}$), *B. forskalii* (Kruskal–Wallis ${\chi }_{3.545, df=6, p=0.738}^{2}$), and *T. granifera* (Kruskal–Wallis ${\chi }_{4.679, df=6, p=0.586}^{2}$) between districts. However, there were statistically significant differences in the abundance of Bivalves (Kruskal–Wallis ${\chi }_{25.44, df=6, p=0.0003}^{2}$), *P. acuta* (Kruskal–Wallis ${\chi }_{27.86, df=6, p=0.00001}^{2}$), *L. natalensis* (Kruskal–Wallis ${\chi }_{14.772, df=6, p=0.022}^{2}$), and *B. tropicus* (Kruskal–Wallis ${\chi }_{23.338, df=6, p=0.0007}^{2}$) between districts. The abundance of *B. tropicus* was significantly lower in eThekwini district compared to iLembe and uMkhanyakude districts (*p-*value < 0.05). See Table S2 for the results of the multiple pairwise comparison between districts.

**Table 1:** Spatial distribution and abundance of intermediate and non-intermediate host snails for schistosomiasis collected in 7 districts of KwaZulu-Natal from September 2020 to August 2021.

District	*Bulinus globosus*	*Biomphlaria pfeifferi*	*Bulinus tropicus*	*Bulinus forskalii*	*Lymnaea natalensis*	*Tarebia granifera*	Bivalves	*Physa acuta*
Ugu	97	482	1126	12	245	661	348	688
eThekwini	174	5	443	11	446	269	81	677
uMkhanyakude	131	365	0	156	159	3943	32	4
Zululand	301	16	33	0	60	2727	0	0
uMzinyathi	46	0	20	0	27	0	0	0
uThukela	585	261	85	13	212	109	0	30
iLembe	62	1	0	0	46	369	0	200

### Effect of seasons and habitat types on snail abundance

Data in Table S1 indicates seasonality in snail abundance with the highest abundance of freshwater snails in KZN recorded during the post-rainy season followed by cold/dry, rainy, then hot/dry season (Fig. 4). The trend of high abundance of freshwater snails during post-rainy season was also observed among *B. globosus*, *B. pfeifferi*, *B. tropicus* and *T. granifera* species. However, *B. forskalii* and *P. acuta* were more abundant during the cold/ dry season while *L. natalensis* and Bivalves were more abundant during the hot/dry and rainy seasons, respectively. A decrease in the abundance of *B. pfeifferi* and *B. globosus* snails was observed during the hot/dry, rainy, and post rainy seasons compared to the cold/dry season (Table 2). However, this relationship was only significant for *B. globosus* snails found in hot/dry seasons compared to cold/dry seasons (*p-*value < 0.05) (Table 2). In addition, *B. globosus* snails (*p-*value < 0.05) were significantly more abundant in streams than dams (Table 2).

**Table 2:** Effect of season, habitat type, climatic, water physicochemical properties, and non-intermediate host snails for schistosomiasis on the abundance of *B. globosus* and *B. pfeifferi* snails.

Snail species	Variables	Estimates	Confidence Interval	Pr >|z|
*B. globosus*	Intercept	− 8.321	− 14.964 to − 1.948	0.010
	Hot/dry	− 5.073	− 8.568 to − 1.579	**0.004**
	Post-rainy	− 0.449	− 3.233 to 2.335	0.752
	Rainy	− 2.375	− 7.384 to 2.634	0.353
	Stream	3.541	0.544 to 6.538	**0.021**
	Rainfall	0.017	− 0.003 to 0.039	0.102
	MinTemp	0.147	− 0.304 to 0.598	0.524
	MaxTemp	− 0.022	− 0.088 to 0.044	0.520
	DO	− 0.095	− 0.281 to 0.091	0.318
	pH	0.193	− 0.047 to 0.434	0.115
	*T. granifera*	− 0.0007	− 0.007 to 0.006	0.822
	*L. natalensis*	0.033	− 0.002 to 0.068	0.067
	*P. acuta*	− 0.013	− 0.060 to 0.034	0.577
	*B. tropicus*	− 0.022	− 0.051 to 0.007	0.143
	Bivalves	0.002	− 0.017 to 0.021	0.827
*B. pfeifferi*	Intercept	− 13.022	− 36.372 to 10.328	0.274
	Hot/dry	− 4.129	− 8.384 to 0.125	0.057
	Post-rainy	− 2.538	− 5.855 to 0.779	0.134
	Rainy	− 5.030	− 12.446 to 2.386	0.184
	Rainfall	− 0.002	− 0.046 to 0.042	0.918
	MinTemp	0.255	− 0.016 to 0.526	0.065
	MaxTemp	0.481	− 0.427 to 1.389	0.299
	DO	− 0.235	− 0.549 to 0.079	0.143
	pH	0.675	0.177 to 1.173	**0.008**
	*T. granifera*	− 0.004	− 0.009 to 0.002	0.212
	*L. natalensis*	0.018	− 0.008 to 0.045	0.187
	*P. acuta*	− 0.229	− 0.399 to − 0.059	**0.001**
	*B. tropicus*	0.001	− 0.023 to 0.026	0.920

Significant *p-*value (*p-*value < 0.05) are in bold.

### Effect of climatic factors and water physicochemical properties on snail abundance

**Figure 5:**
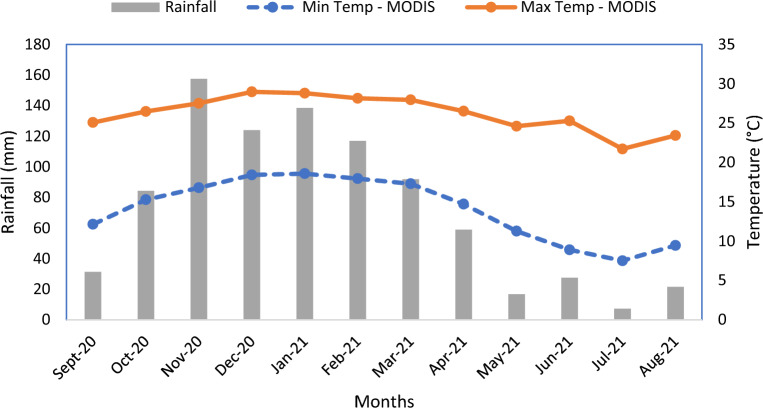
Average monthly rainfall (mm), minimum and maximum land surface temperature (°C) for the study area between September 2020 and August 2021.

The average monthly rainfall (mm), minimum and maximum land surface temperature $(^\circ{\rm C} )$ for the study area between September 2020 and August 2021 is presented in Fig. 5. The highest and least rainfall were recorded in November 2020 and July 2021, respectively. Maximum LST was highest in December 2020 and least in July 2021 (Fig. 4). pH was positively associated with the abundance of *B. pfeifferi* (*p-*value < 0.05) while other climatic factors and water physicochemical properties did not show any statistically significant association with *B. pfeifferi*. None of the climatic factors and water physicochemical properties showed a statistically significant association with the abundance of *B. globosus* (Table 2).

### Effect of other snail species on the distribution and abundance of *B. globosus* and *B. pfeifferi* snails

*B. globosus* and *B. pfeifferi* had a negative relationship with the abundance of both *P. acuta* and *T. granifera*. However, this relationship was only significant between the abundance of *P. acuta* and *B. pfeifferi* (*p-*value < 0.05) (Table 2).

### Spatial and seasonal infection rates of *B. globosus* and *B. pfeifferi* snails

The highest infection rate of *B. globosus* snails was recorded in uMkhanyakude district (31%) followed by iLembe district (3%), Ugu district (1%), uThukela (1%) and Zululand district (1%). *B. globosus* snails found in eThekwini and uMzinyathi districts did not shed human cercariae (Table 3). Of all the *B. pfeifferi* snails found in the study area, schistosome infections were only found in uMkhanyakude district where the infection rate was 3% (Table 3). Although different infection rates were recorded in each district (Table 3), these differences were not statistically significant between districts for both *B. globosus* (Kruskal–Wallis ${\chi }_{6.599, df=6, p=0.340}^{2}$), and *B. pfeifferi* (Kruskal–Wallis ${\chi }_{7.388, df=6, p=0.287}^{2}$).

**Table 3:** Number of *B. globosus* and *B. pfeifferi* collected and infection rates by districts.

District	Number of *B. globosus* collected	Number of infected *B. globosus* snails. (Infection rate (%))	Number of *B. pfeifferi* collected	Number of infected *B. pfeifferi* snails. (Infection rate (%))
Ugu	97	1(1)	482	0 (0)
eThekwini	174	0 (0)	5	0 (0)
uMkhanyakude	131	40 (31)	365	10 (3)
Zululand	301	3 (1)	16	0 (0)
uMzinyathi	46	0 (0)	0	0 (0)
uThukela	585	3 (1)	261	0 (0)
iLembe	62	2 (3)	1	0 (0)

**Figure 6:**
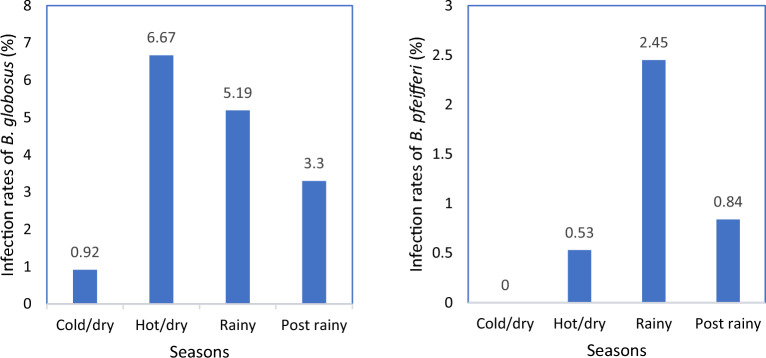
Infection rates of *B. globosus* and *B. pfeifferi* by seasons.

*B. globosus* collected during the study period shed mammalian cercariae across the four seasons while *B. pfeifferi* snails shed mammalian cercariae in three seasons excluding the cold/dry season (Fig. 6). The infection rates for *B. globosus* snails (n = 49, 3.5%) were higher compared to that of *B. pfeifferi* snails (n = 10, 0.8%). The highest snail infection rate of *B. globosus* and *B. pfeifferi* snails were recorded in the hot/dry (6.67%) and rainy seasons (2.45%), respectively as shown in Fig. 6. Rainfall and pH showed a statistically significant negative association with abundance of shedding *B. globosus* based on the negative binomial GLMM (Table 4). There was a statistically significant increase in the infection rates of *B. globosus* in the post rainy season compared to the cold/dry season (*p-*value < 0.05) (Table 4). On the other hand, climatic factors, water physicochemical parameters, and seasons did not affect the infection rates of *B. pfeifferi* snails.

**Table 4:** Effect of season, climatic and water physicochemical properties on the infection rates of *B. globosus* and *B. pfeifferi* snails.

Snail species	Variables	Estimates	Confidence interval	Pr( >|z|)
*B. globosus*	Intercept	− 3.502	− 13.499 to 6.494	0.492
	Hot/dry	1.922	− 2.856 to 6.699	0.431
	Post-rainy	4.196	0.320 to 8.071	**0.034**
	Rainy	4.438	− 2.926 to 11.803	0.238
	Rainfall	− 0.057	− 0.113 to − 0.001	**0.048**
	MinTemp	0.168	− 0.206 to 0.542	0.379
	Max Temp	0.065	− 0.288 to 0.417	0.720
	DO	0.383	− 0.187 to 0.953	0.188
	pH	− 0.774	− 1.545 to − 0.004	**0.049**
*B. pfeifferi*	Intercept	− 147.899	− 409.783 to 113.984	0.268
	Post-rainy	2.681	− 5.499 to 10.862	0.521
	Rainy	− 4.395	− 38.945 to 30.154	0.803
	Rainfall	− 0.124	− 0.406 to 0.159	0.392
	MinTemp	3.263	− 6.517 to 13.043	0.513
	MaxTemp	3.180	− 2.108 to 8.468	0.238
	DO	− 0.624	− 3.062 to 1.814	0.616
	pH	0.797	− 1.930 to 3.524	0.567

Significant *p-*value (*p-*value < 0.05) are in bold.

## Discussion

In this study, a longitudinal survey was conducted between September 2020 to August 2021 at 79 sites in 7 districts in KZN province, South Africa to determine the distribution and abundance of freshwater snails and how certain parameters like climate, water physicochemical, habitats, seasons and abundance of non-human schistosomiasis transmitting intermediate host snails influence the distribution, abundance, and infection rates of human schistosomiasis intermediate host snails. Eight freshwater snail species including those that transmit schistosomiasis were identified. Although, *P. acuta*^20^, *T. granifera*^21^ and Bivalve are disease hosts in other countries, it remains speculative in South Africa as they have no role in the transmission of any significant snail-borne disease.

A statistically significant positive relationship was observed between the abundance of *B. globosus* and the habitat stream. Although, *B. globosus* are found in a wide range of habitats ranging from rivers, streams, dams, and seasonal ponds. Most of the *B. globosus* snails in our study were found in streams compared to dams which corroborates findings from previous research^6^. This could be attributed to their ability to tolerate moderate pollution and preference for habitat with clear water, sandy and gravel substrates compared to dams and ponds with muddy substrates^22,23^. However, Woolhouse and Chandiwana^24^ opined that since *B. globosus* are found in a wide range of habitats, climatic factors such as rainfall and temperature are rather important factors that affects its abundance and dynamics.

Although rainfall did not have a statistically significant relationship with the abundance of *B. globosus* in our study, a statistically significant relationship was observed between the rainfall and the infection rates of *B. globosus* (*p-*value < 0.05). Several studies^6,24,25^ have attempted to describe the relationship between snail abundance, distribution, infection rates and rainfall. Rainfall is a very important parameter that affects snail distribution and abundance in different ways. This is because snails need water to grow, reproduce and survive maximally but too much water leads to reduction in the snail population due to flooding and washing away of the snails since they are not able to attach themselves to rocks and plants. Manyangadze, et al.^6^ explained that snail population could decrease when water velocity exceeds 0.3 m/s. Although, we did not have measurements for water velocity in our study, it could suggest that the snail species that had a significant negative relationship with rainfall had a water velocity greater than 0.3 m/s and vice versa for the snail species that has a significant positive relationship with rainfall. High rainfall results in reduction in snail abundance and establishment of new sites downstream for dispersed snails while low rainfall during the dry seasons may lead to reduction in snail abundance due to drought^24,26^.

Variations were observed in the abundance of *B. globosus* and *B. pfeifferi* across the study seasons. The highest abundance of *B. globosus* was recorded during the post-rainy season, followed by the rainy, cold/dry, and hot/dry seasons. The high *B. globosus* counts recorded in the post-rainy season could be attributed to the streams and dams having sufficient water and appropriate water and air temperatures that support snail reproduction, growth, and survival. Lower *B. globosus* abundance was recorded in the rainy season compared to the post-rainy season, this could be because of the high-water flow due to rainfall that may have resulted to washing away of the snails due to flooding of the rivers and dams. In addition, the lowest *B. globosus* counts recorded in the hot/dry season could be due to low rainfall that resulted in drought. There was a statistically significant decrease in the abundance of *B. globosus* in the hot/dry season compared to cold/dry season and could be attributed to the high temperatures and very low rainfalls experienced in the hot/dry season which may have resulted in most of the rivers and dams being dry, and the snails aestivating^25,27^. Our findings in terms of high *B. globosus* abundance in the post-rainy and rainy seasons and decrease in abundance in the cold/dry season corroborate the finding by Woolhouse and Chandiwana^24^. In contrast to our finding, *B. globosus* abundance increased in the hot/dry season^6,24^ and decrease during the rainy and post-rainy seasons^6^. *B. pfeifferi* was more abundant during the post-rainy and cold/dry seasons compared to other seasons. Similar observations were reported by Bakhoum, et al.^28^, Woolhouse^26^ and Manyangadze, et al.^6^.

*B. globosus* and *B. pfeifferi* snails are the intermediate host snails for transmitting *S. haematobium* and *S. mansoni*, respectively that were found in the study area. Seasonal variations were observed in the infection rates of the snails. This could be explained by the positive relationship between temperature and infectivity, increase in miracidial input and infections^29,30^. The high and no infection rates of *B. pfeifferi* snails in the rainy and cold/dry seasons, respectively could be explained by the low temperatures observed during the cold/dry season. In addition, since majority of the rivers and dams are dry due to lack of rainfall during the dry season, less people visit the waterbodies and there is less miracidial input from faeces deposited that could be washed into the waterbody by rains^31^. There was a statistically significant increase in the number of *B. globosus* that shed cercariae during the post-rainy season compared to the cold/dry season. These findings corroborate the observation by Augusto, et al.^32^ who reported higher infection rates of *B. globosus* shedding *S. haematobium* cercariae during the rainy season and lower infection rates during the dry season. This could be due to the availability of water in the rivers because of the rains that attracts more people leading to more miracidial input.

*B. globosus* snails were found to be infected with *S. haematobium* in Ugu, uMkhanyakude, Zululand, uThukela, and iLembe districts with the highest infection rate recorded in uMkhanyakude district. On the other hand, *B. pfeifferi* snails were found in 6 of the 7 districts where snail sampling was carried out. However, infected *B. pfeifferi* snails were found in uMkhanyakude district only. We recorded higher infection rates of 31% and 3% for *B. globosus* and *B. pfeifferi*, respectively compared to the study carried out by Manyangadze, et al.^6^ in the same district where they recorded infection rates of 8.9% and 0.1% for *B. globosus* and *B. pfeifferi*, respectively. These high infection rates could be explained by climatic changes that could have taken place due to global warming effects and, we sampled from a wider range of waterbodies across uMkhanyakude district compared to the micro-geographical study that took place in Ndumo. Contrariwise, the 2017 report from the department of health^33^ reported zero prevalence of *S. mansoni* in uMkhanyakude district, but our study reports contrary. Finally, uMkhanyakude is one of the most rural and impoverished districts in KZN and could explain the reason for the high infection rates of *B. globosus* and *B. pfeifferi* as well as the temperature and rainfall being optimal for schistosomiasis transmission.

There was no significant relationship between pH and the abundance of *B. globosus* snails, and this has been reported before by Opisa, et al.^34^. However, pH had a negative statistically significant relationship with the infection rates of *B. globosus* (*p-*value < 0.05)*,* which suggests that lower pH (more acidic < 7) value is associated with an increase in the infection rates of *B. globosus* snails. This is similar to the report by Levitz, et al.^35^ but contrary to report from Bilkovic and Walby^36^ where lower pH values resulted in significantly lower growth rates and fecundity. However, other studies have argued that pH is not an important parameter that influences snail distribution, abundance, and infection rates. The pH levels of sites where infected *B. globosus* snails were found ranged from 6.8 to 8.2. The complexities surrounding the interpretation of the biological significance of pH measurements on snail distribution, abundance, and infection rates was noted by Brown^22^.

The abundance of *P. acuta* and *T. granifera* had a negative relationship on the abundance of both *B. globosus* and *B. pfeifferi*. However, this negative relationship was only significant between *P. acuta* and *B. pfeifferi* (*p-*value < 0.05). This could be attributed to the rapid generation time and high reproductive rate in *P. acuta* leading to increase in population that outnumbers *B. pfeifferi*. *P. acuta* is an invasive species capable of replacing snail species within the genera *Bulinus spp* and *Biomphalaria spp* responsible for the transmission of urogenital and intestinal schistosomiasis^37^. In addition, it is speculated that *P. acuta* secretes some form of chemical inhibitors where *B. pfeifferi* is present as *B. pfeifferi* growth rates and egg output declines^37^. Competitive snail species have been used as biological control to successfully displace native snails that transmit schistosomiasis. In Mozambique, *P. acuta* snails were used to eliminate the native host snail that causes human schistosomiasis^38^.

## Conclusion

The study revealed a seasonal difference in the distribution and abundance of the snails identified, with the highest abundance occurring in the post-rainy season. Our findings indicate that there is active schistosomiasis transmission in the study area. The impact of seasons on the shedding times of *B. globosus* and *B. pfeifferi* snails serving as intermediate host snails for *S. haematobium* and *S. mansoni*, respectively, provides information on the timing to administer preventive treatment and carry out snail control. Rainfall, seasons, habitats, and water pH were identified as parameters that affect snail distribution, abundance, and infection rates. The negative association between the abundance of *P. acuta* and *B. pfeifferi* suggest a possible control mechanism for schistosomiasis.

Although our study provided valuable results, a specific limitation is that our data was collected 4 times a year, once in every season which could make it difficult to draw some conclusions. Collecting data more than once every season for a minimum of a year will account for within season variability thereby cushioning the effect of extreme climatic conditions such as rainfall and temperature. In addition, snail species and cercariae were identified morphologically which are less precise compared to using molecular markers and diagnostics.

## Supplementary Information

Supplementary Information 1.

Supplementary Information 2.

## Data Availability

The data generated and/or analysed during the current study are not publicly available because it is the intellectual property of the University of KwaZulu-Natal but are available from the corresponding author on reasonable request.
