# Supplementary material for: Spatial and seasonal distribution of human schistosomiasis intermediate host snails and their interactions with other freshwater snails in 7 districts of KwaZulu-Natal province, South Africa
Source: Sci Rep. 2023 May 15;13:7845. doi: 10.1038/s41598-023-34122-x (PMC10185499; doi:10.1038/s41598-023-34122-x)
Supplement: Supplementary file 1 — Supplementary Information 1. [file 41598_2023_34122_MOESM1_ESM.doc]

Table S1. Seasonal distribution and abundance of intermediate host and non-intermediate host snails for human schistosomiasis at sampling sites during 4 seasons.

| Months | Seasons | Intermediate host snails for human schistosomiasis | | | Other snail species | | | | | | | Grand  Total |
| --- | --- | --- | --- | --- | --- | --- | --- | --- | --- | --- | --- | --- |
|  |  | *B. globosus* | *B. pfeifferi* | Total | *B. tropicus* | *B. forskalii* | *L. natalensis* | *T. granifera* | Bivalves | *P. acuta* | Total |  |
| 2020 September-November | Hot and dry | 30 | 189 | 219 | 55 | 0 | 401 | 1114 | 35 | 92 | 1697 | 1916 |
| 2020 December-2021 February | Rainy | 366 | 245 | 611 | 592 | 18 | 94 | 1733 | 370 | 10 | 2817 | 3482 |
| 2021 March-May | Post-rainy | 783 | 359 | 1142 | 698 | 17 | 353 | 3801 | 40 | 545 | 5454 | 6596 |
| 2021 June-August | Cold and dry | 217 | 337 | 554 | 377 | 160 | 347 | 1430 | 16 | 932 | 3262 | 3816 |
| Total |  | 1396 | 1130 | 2526 | 1722 | 195 | 1195 | 8078 | 461 | 1579 | 13230 | 15810 |
