# Supplementary material for: Spatial and seasonal distribution of human schistosomiasis intermediate host snails and their interactions with other freshwater snails in 7 districts of KwaZulu-Natal province, South Africa
Source: Sci Rep. 2023 May 15;13:7845. doi: 10.1038/s41598-023-34122-x (PMC10185499; doi:10.1038/s41598-023-34122-x)
Supplement: Supplementary file 2 — Supplementary Information 2. [file 41598_2023_34122_MOESM2_ESM.docx]

S2: Result of multiple pair-wise comparison between districts.

| Snail species | District 1 | District 2 | Statistics | p-value |
| --- | --- | --- | --- | --- |
| Bivalves | eThekwini | Ugu | 3.533 | **0.009** |
|  | iLembe | Ugu | 3.910 | **0.002** |
|  | Ugu | uMkhanyakude | -3.693 | **0.005** |
|  | Ugu | uMzinyathi | -3.777 | **0.003** |
|  | Ugu | uThukela | -4.283 | **0.0004** |
|  | Ugu | Zululand | -4.283 | **0.0004** |
| *P. acuta* | eThekwini | uMkhanyakude | -3.828 | **0.003** |
|  | eThekwini | uMzinyathi | -3.214 | **0.024** |
|  | eThekwini | Zululand | -3.798 | **0.003** |
| *B. tropicus* | eThekwini | iLembe | -3.661 | **0.046** |
|  | eThekwini | uMkhanyakude | -3.782 | **0.003** |
|  | uMkhanyakude | uThukela | 3.120 | **0.038** |
| *L. natalensis* | eThekwini | uMkhanyakude | -2.422 | **0.015** |
|  | iLembe | Ugu | 1.967 | **0.049** |
|  | Ugu | uMkhanyakude | -2.624 | **0.009** |
|  | Ugu | Zululand | -2.121 | **0.034** |
|  | uMkhanyakude | uThukela | 2.532 | **0.011** |

Significant p-value (p-value < 0.05) are in bold.
